# Atlas pre-selection strategies to enhance the efficiency and accuracy of multi-atlas brain segmentation tools

**DOI:** 10.1371/journal.pone.0200294

**Published:** 2018-07-27

**Authors:** Chenfei Ye, Ting Ma, Dan Wu, Can Ceritoglu, Michael I. Miller, Susumu Mori

**Affiliations:** 1 Department of Electronics and Information, Harbin Institute of Technology Shenzhen Graduate School, Shenzhen, Guangdong Province, China; 2 National Clinical Research Center for Geriatric Disorders, Beijing, China; 3 The Russell H. Morgan Department of Radiology and Radiological Science, The Johns Hopkins University School of Medicine, Baltimore, MD, United States of America; 4 Center for Imaging Science, The Johns Hopkins University School of Medicine, Baltimore, MD, United States of America; 5 F.M. Kirby Research Center for Functional Brain Imaging, Kennedy Krieger Institute, Baltimore, MD, United States of America; Pennsylvania State Hershey College of Medicine, UNITED STATES

## Abstract

Multi-atlas brain segmentation of human brain MR images allows quantification research in structural neuroimaging. To achieve high accuracy and computational efficiency of segmentation relies on a custom subset of atlases for each target subject. However, the criterion for atlas pre-selection remains an open question. In this study, two atlas pre-selection approaches based on location-based feature matching were proposed and compared to random and mutual information-based methods using a database of 47 atlases. A varying number of atlases ranked top with hierarchical structural granularity were compared using Dice overlap. The results indicated that the proposed 4L approach consistently led to the highest level of accuracy at a given number of employed atlases in both adult and geriatric populations. In addition, the proposed two methods (4L and LV) can reduce 20 times computational time compared with the stereotypical mutual information-based method. Our pre-selection strategy would provide better segmentation performance in terms of both accuracy and efficiency. The proposed atlas pre-selection will be further implemented into our online automatic brain image segmentation system (www.mricloud.org).

## Introduction

Quantitative analysis of brain MR images has been a subject of active research during the past two decades, in which, the voxel-based analysis (VBA) became one of the most widely used approaches. In this approach, all subject images are registered to a single anatomical template and the morphological parameters or signal intensity values are statistically evaluated in a voxel-by-voxel manner. With 1x1x1mm image resolution, a typical brain consists of more than 1 million voxels. Because voxels are the minimum unit to define locations, the VBA guarantees the maximum amount of location information in a completely reproducible and automated manner. Once potential anatomical abnormalities are found by image analyses, the correlation of certain anatomical features with clinical outcomes would be an important next step. However, such a correlation between 3D imaging domain and non-imaging clinical features would face a considerable challenge. Namely, more than 1 million independent, and potentially noisy, measurements would pose a considerable burden in subsequent analyses. At some point, a smart data reduction in the anatomical domain becomes a crucial step.

One approach is the whole-brain brain segmentation, in which the multiple anatomical structures are defined automatically. For example, SPM has been widely used to decompose the brain into the gray matter, white matter, and CSF. FSL and FreeSurfer are widely used to segment the brain into various pre-defined regions. These approaches can be considered as a smart anatomical filter to join voxels into a small number of structural representations, potentially solving the “curse of dimensionality” and facilitate the accuracy of the subsequent correlational analyses with clinical outcomes.

In the past decade, we have witnessed a rapid advancement of brain segmentation technologies, stimulated by multiple atlas fusion algorithms. In this approach, a large number of atlases with pre-defined structures are registered to each subject image. Then the structural labels are casted from the atlases to the subject, followed by a fusion process to achieve the best estimation of the structural boundaries. Many atlas fusion algorithms have been proposed, which take not only the label locations but also the intensity information into consideration. While the improvement of fusion algorithm has been a target of active research to achieve better accuracy, the quality of the results also relies heavily on the atlases. The framework of the multi-atlas approach is similar to machine learning and the atlas library serves as a teaching file dictating which structure should be where.

The multiple atlas library, which serves as the knowledge database, poses interesting, yet difficult questions. Such questions include, how many atlases are needed, should age be matched and if so how precise it should be, and are images from healthy subjects appropriate to accurately segment patients with anatomical abnormalities? The atlas library also needs to include structural label files, which are transferred from the atlases to the test image followed by image-to-image registration. Then the pre-defined labels are used as a spatial filter to group voxels that belong to the same structures. For example, more than 1 million voxels are reduced to 286 structures in our atlases. However, there are numerous ways to define structures and we cannot always assume the available labels are the most appropriate to characterize the pathology of interest. In addition, the structures need to be defined in all atlases with high accuracy and consistent anatomical criteria.

We believe that there are no generic answers to all these questions, but it should be possible to develop a general framework to achieve better segmentation accuracy. For example, we can safely assume that the availability of atlases that share similar anatomical features to a given test image would increase the segmentation accuracy. Obviously, we do not want 80-years-old atlases to segment pediatric data. We also do not want to use 1,000s of atlases to segment one image for the sake of efficiency. This naturally leads to the idea of atlas pre-selection to maximize the computational efficiency with minimized accuracy loss. One computationally straightforward approach would be to match non-image attributes such as age and disease type. Another approach, which is not mutually exclusive to the non-image attributes, is to match the global anatomical features. The global features may include both the brain tissue itself and the extracranial structures such as dura, bone marrow, and skin. As a measure of image global features, mutual information (MI) [1] was employed in multi-atlas segmentation. It is shown that MI-based atlas pre-selection enhanced brain segmentation performance [2, 3]. However, there was also a report indicating that the mutual information based image similarity between atlas and target image is not directly related to the final segmentation performance, especially when small sets of atlases are selected [4].

Our laboratory has been developing a brain atlas library including fully segmented atlases. As the atlas inventory grows, the importance of the atlas pre-selection scheme increases. One merit of the atlases in our database is that they have unique hierarchical structural definitions so that each atlas contains different representation of the brain with varied granularity. Our previous study [5] has indicated an atlas pre-selection strategy based on a dynamic age-matching approach would improve accuracy of multi-atlas brain segmentation. This demographic criterion of atlas pre-selection hypothesized brains with similar ages share similar anatomical sizes and shapes, which is not always true especially for brains with pathology. In this paper, we tested different atlas pre-selection strategies based on image-based similarity criterion to evaluate the brain segmentation accuracy and efficiency at different granularity levels and for different ages.

We delivered our multi-atlas segmentation pipeline via the MRICloud platform (https://braingps.MRICloud.org). In this pipeline, the group of atlases were initially propagated to the target image respectively, with the likelihoods fused to form a consensus log-posterior from which the estimated target segmentations are derived [6]. For the implementation on MRICloud, segmentation result can be obtained through 3 steps, i.e. preprocessing, atlas registration, and label fusion. Given a segmentation job submitted to MRICloud, the target image is first normalized into MNI space, followed by an atlas registration and label fusion to generate a coarse 7-label segmentation, by fast algorithms. Then large deformation diffeomorphic metric mapping (LDDMM) [7] is applied to map atlases to target image, followed by the likelihood-fusion using expectation-maximization (EM) algorithm to give an elaborate 286-label segmentation result [8]. The complexity of LDDMM algorithm is a major concern for the computation of MRICloud. As each atlas provides unique brain structure, increasing the number of atlases would provide a better representation of anatomical structure. As a result, computation of the MRICloud across multiple atlases becomes a significant bottleneck which increases with the enrichment of the atlas database. Therefore, current atlas pre-selection is proposed as a step after the preprocessing step based on the 7-label coarse segmentation result.

## Methods and materials

### Multi-atlas brain segmentation pipeline

A query image is segmented by multiple atlases in two steps, image registration and label fusion, where the atlases are pre-defined by expert annotations. The anatomical information from multiple atlases is transferred across the whole brain by small and large global deformation. Thus target images can be segmented by establishing the voxel-wise correspondence relationship, where the labels get mapped through such deformation [9]. In our multi-atlas brain segmentation pipeline, the target image was first aligned to MNI space [10] using affine registration, followed by likelihood fusion process based on expectation-maximization (EM) [8] to generate a coarse 7-label segmentation result. The 7 labels include the CSF space, the gray matter, the white matter, the ventricles, tissues in the skull base (optic chiasm, optic nerves, pituitary gland, and other tissues which have similar intensities as the adjacent brain tissues), skin (includes lipid layer), and the remaining space. This initial step takes approximately 1~2 mins using the cloud system. Based on the initial 7-label segmentation, the brain tissues were defined and intensity corrections (field bias correction within each subject and atlas-image histogram matching) were performed, the atlases were registered to the target image using LDDMM [7]. The resultant transformation matrices were then applied to the segmentation files, which were then combined using the multi-atlas likelihood fusion (MALF) algorithms [8,11], generating the final 286 segments. More detail descriptions about our pipeline can be found in our previous publications [8, 9,11].

### Atlases

**Fig 1:**
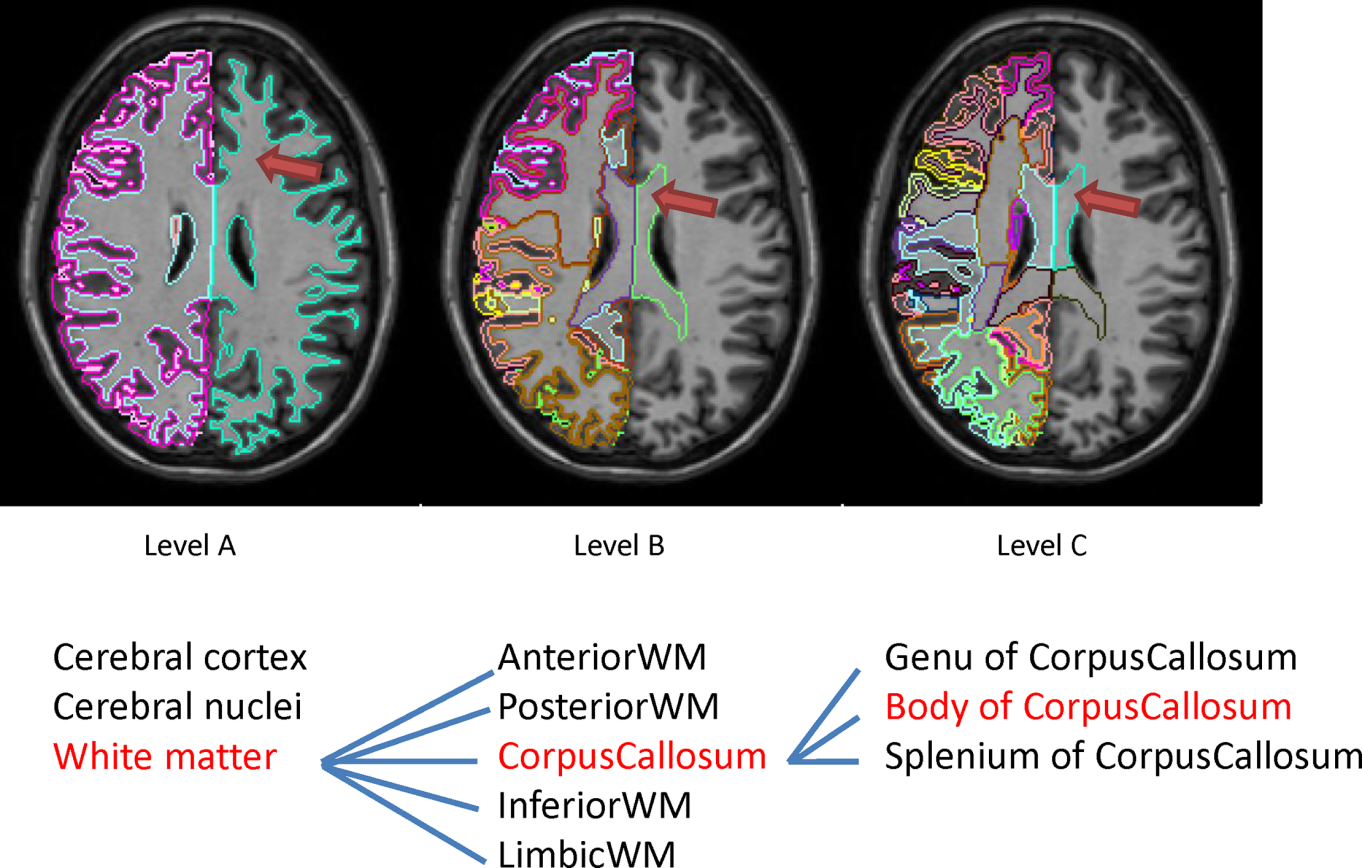
Illustration of 3 ontology levels of the segmentation results from our multi-atlas brain segmentation pipeline within the same subject. The structures defined by red name are indicated in the images in red arrows. The name of each structure is listed in Table 1.

The atlas library consists of MPRAGE images and segmentation files (Version 7.1) that define 7 whole-head coarse labels and 286 brain structures. The details of this atlas library are described in [5]. The anatomical criteria were based on our single-subject atlas [12]. The minimum definable units of the structures are usually based on available MR contrasts; it is difficult to subdivide a structure if its internal structures do not have unique contrasts. One of the uniqueness of our atlases is that 213 additional brain structures are defined by combining the 286 structural units and creating superstructures [13] in multiple hierarchical levels. For example, the hippocampus belongs to the limbic system, together with other related structures such as the amygdala, parahippocampal gyrus, and the entorhinal cortex, which are then included to the total gray matter. The ontology-based hierarchical relationship follows those by [14,15] as much as possible. This hierarchical and anatomy-based voxel-grouping filter can examine the regional specificity of observed abnormalities and detection sensitivity at multiple granularity levels [16]. Fig 1 shows pictorial presentations of the hierarchical relationship. In the current study, the atlas database contains 47 T1 images from subjects 22 to 86 years old, consisting two age groups: geriatric (age = 71.3 ± 7.0) and adult (age = 35.6 ± 11.4). In order to cover the wide range of anatomical phenotypes in the multi-atlas based segmentation, subjects with potential brain atrophy or AD are also included (n = 6). To test pre-selection methods, we randomly selected 20 atlases in the database (10 from each age group) as the target images following a leave-one-out cross validation. Part of the Atlases can be accessed in osf.io/ngewh.

### Atlas pre-selection

It has been indicated that the segmentation accuracy increases as the number of atlas sets increase [3]. Meanwhile, the computational cost grows proportionally as the number of the atlases increases. On the other hand, if there are only 10 population-representative atlases and the target image has, for example, enlarged ventricles with an additional 50 percentile volume of average, none of the atlas may have similar anatomy. If there are 100 atlases, a fusion algorithm may properly give weight to 10 atlases with similar ventricle shapes. Of course, we can argue better image registration tools or smarter fusion algorithms can effectively diminish the adverse effect of anatomical minority, but enrichment of atlases with similar anatomical features through atlas pre-selection remains an interesting research topic.

The main principle of atlas pre-selection is to rank all atlases in the database according to an agreement with target image, so that the top-ranked atlases are selected for subsequent image registration and fusion processes. In previous studies, MI was adopted as a measure for the atlas ranking and improved accuracy by the atlas pre-selection was observed compared to the randomly selected atlases especially when the number of employed atlases decreased [2, 3]. Machine learning was also proposed for the atlas pre-selection [4].

The aforementioned methods are aiming at selecting best matching atlases to the target image based on voxel-by-voxel features. A segment-by-segment analysis can be an alternative approach to perform anatomical feature matching, in which more than 1 million voxels from a whole brain are aggregated to a small set of anatomical representations and anatomical correlation is performed. This approach, however, contains a recursive problem. Anatomical segmentation is not available until a segmentation tool is completed when choosing proper atlases for a segmentation tool. For the design of a segmentation-based atlas pre-selection method, we need an efficient initial estimation of brain segmentation prior to a more thorough segmentation algorithm.

**Fig 2:**
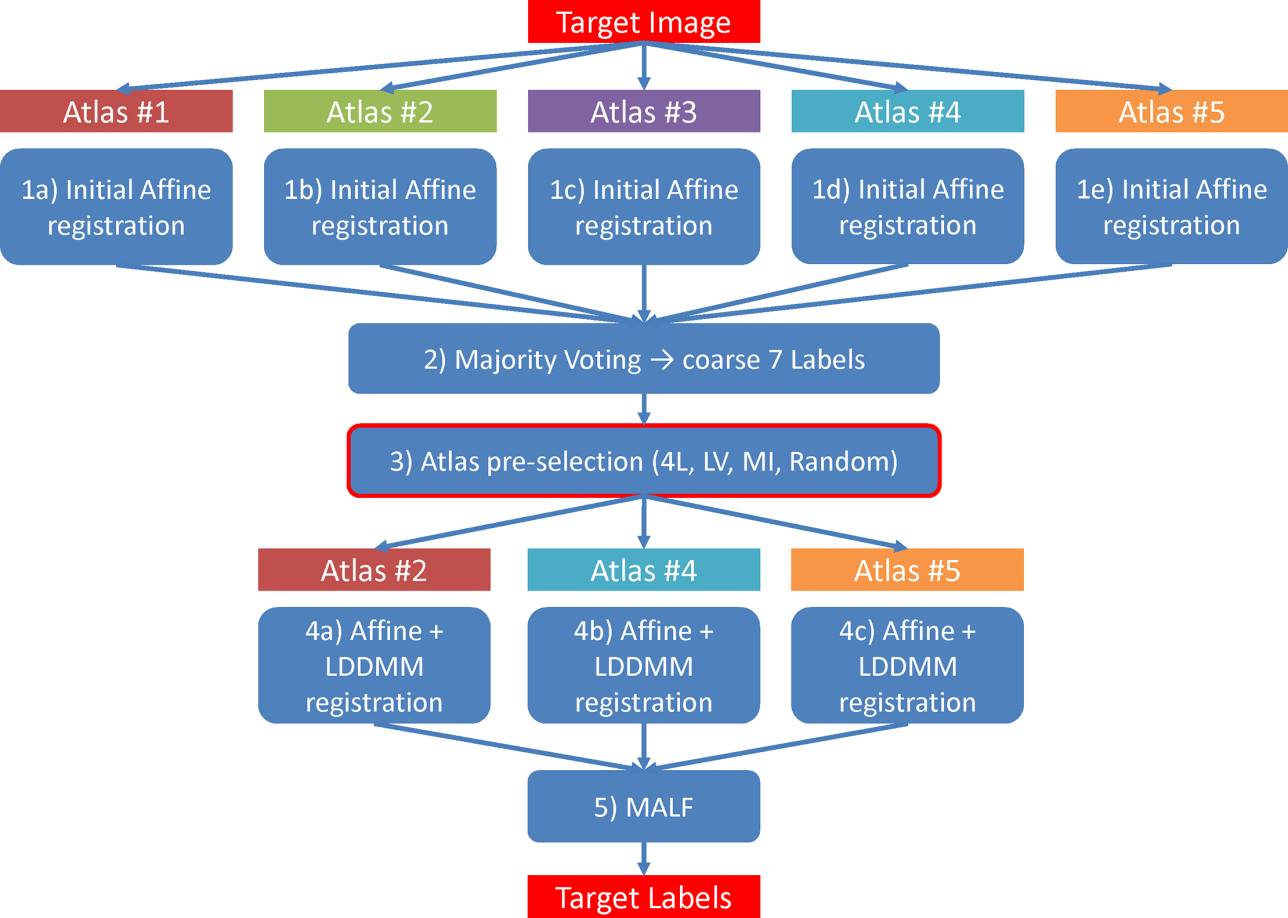
The schematic workflow of the segmentation pipeline. Given 5 atlases in use, 3 atlases were selected through the pre-selection step to estimate the final segmentation labels of the target image.

In this paper, we aim to develop a fully automated segmentation tool that starts with raw (unprocessed) MR images. We utilized the initial 7-label segmentation in our pipeline and tested the efficacy of segmentation-based approaches. The schematic workflow of the segmentation pipeline is illustrated in Fig 2. The performances with different pre-selection approaches in this study were compared offline in a unified PC environment with Intel Core i5 and 8 GB RAM. The details of our pre-selection protocol can be accessed at dx.doi.org/10.17504/protocols.io.pybdpsn.

**Fig 3:**
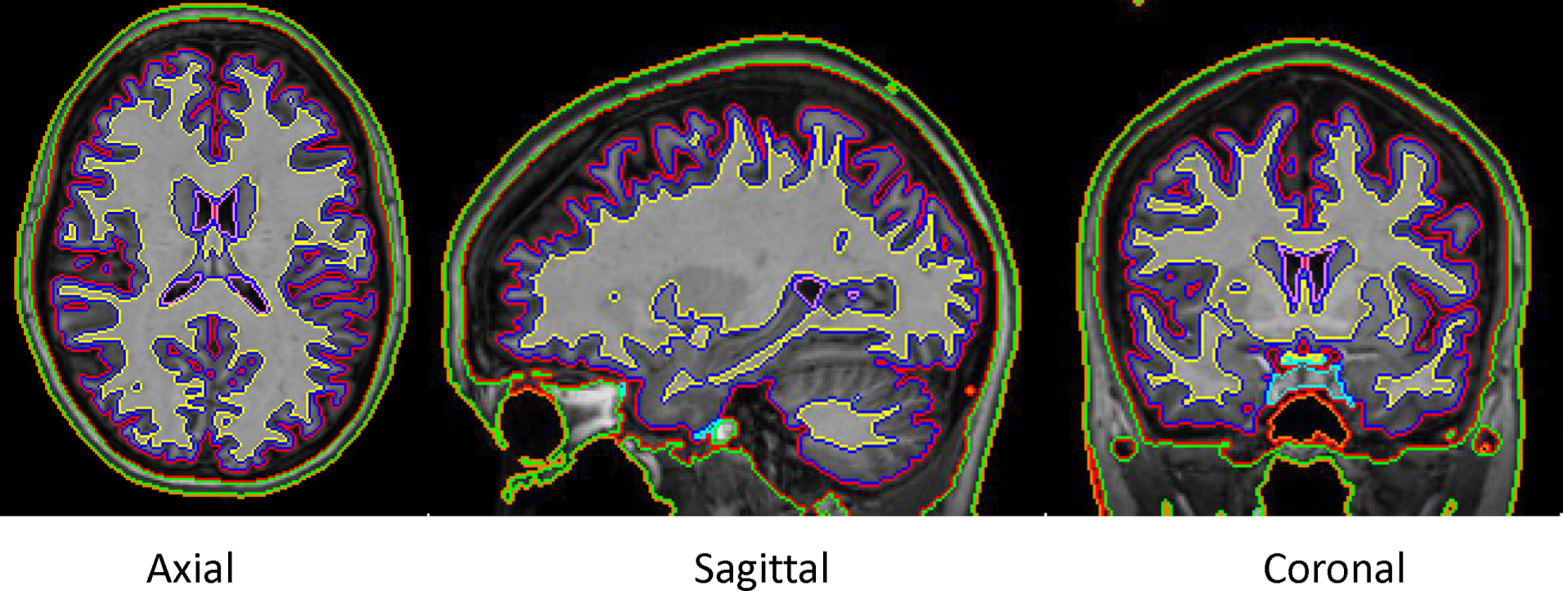
The coarse 7-label segmentation for a single subject. The 7 labels include the CSF space with red boundary, the gray matter with blue boundary, the white matter with yellow boundary, the ventricles with pink boundary, tissues in the skull base (optic chiasm, optic nerves, pituitary gland, and other tissues which have similar intensities as the adjacent brain tissues) with cyan boundary, skin (includes lipid layer) with green boundary, and the remaining space with orange boundary.

The segmentation-based approach was based on Dice overlap of representative ROIs in the coarse 7-label segmentation map (Fig 3). Dice is a statistical validation metric to evaluate the overlapping accuracy of paired structural regions [17]. It is commonly used to evaluate the agreement of segmentations between an automated segmentation estimate and the manual one. Its coefficient is given by

$$Dice(W_{i},W_{j})=\frac{2|W_{i}\cap W_{j}|}{\left|W_{i}|+|W_{j}\right|}$$ (1)

where W_i_ and W_j_ represent the regions of the labeled region for comparison. In other words, Dice measures the ratio between the intersection area and the union area of the paired structural regions.

Among the 7 labels, the 4 labels (4L) related to the gray matter, the white matter, the lateral ventricle, and the surrounding CSF space were adopted to calculate the Dice between the target image and each atlas, which results in a 4L-based atlas pre-selection. We also tested one-label approach using the lateral ventricle (LV-based). Sub-atlas sets were created by pre-selecting the top 5, 10, 15, 20, 25 atlases using the Dice measures. For comparison, we also tested MI-based approach using voxel intensity information from the whole brain image. The MI calculation was based on the entropy of the voxel intensity distribution of a volume, which is calculated via

$$H(I)=-\sum_{i\in I}P(i)\log P(i)$$ (2)

where *P*(*i*) is the probability distribution of the voxel intensities of volume *I*. If *H*(*I*_*A*_), *H*(*I*_*B*_) are used to denote the marginal entropies of volumes *I*_*A*_ and *I*_*B*_, and *H*(*I*_*A*_,*I*_*B*_) denotes their joint entropy, then the MI, which expresses the amount of information that volume *I*_*A*_ contains about volume *I*_*B*_, is given by

$$\mathrm{MI}(I_{A},I_{B})=(H(I_{A})+H(I_{B}))/H(I_{A},I_{B})$$ (3)

The mutual entropy of the two volumes is similarly defined in terms of the joint probability distribution of the voxel intensities, summed over all overlapping voxel pairs [18].

Randomly selected subsets with 5, 10, 15, 20, 25 atlases were also generated for each target image for comparison, where the resulted segmentation is taken as the benchmark reference. In brief, each target image is segmented by a total 20 atlas subsets, including the randomly selected and the top 5, 10, 15, 20, 25 selected based on 4L-, LV-, and MI-based methods. The segmentation task is accomplished by combining each atlas selection method with our multi-atlas brain segmentation pipeline.

### Evaluation of segmentation accuracy

The efficacy of the atlas pre-selection was measured by the segmentation accuracy. Because all data were extracted from an atlas library with manually corrected segmentation files, the automated segmentation results can be directly compared to the available atlas segmentation files. The agreement between these two segmentation files was also measured by Dice overlap. Among the multiple granularity levels, we extracted a total of 63 structures (4 structures from Level A, 26 from Level B, and 33 from Level C in Fig 1), which are clearly definable using the MPRAGE contrasts and widely accepted for both anatomical and clinical scenarios (Table 1). The hierarchical relationships of different granularity levels are listed in S1 Table.

**Table 1:** ROIs for evaluation test under Level A, Level B and Level C.

Level A	Level B	Level C
CerebralCortex_L	Frontal_L	Amygdala_L
CerebralCortex_R	Frontal_R	Amygdala_R
WhiteMatter_L	Parietal_L	Hippocampus_L
WhiteMatter_R	Parietal_R	Hippocampu_R
	Temporal_L	Caudate_L
	Temporal_R	Caudate_R
	Limbic_L	Putamen_L
	Limbic_R	Putamen_R
	Occipital_L	Globus pallidus_L
	Occipital_R	Globus pallidus_R
	midbrain_L	Thalamus_L
	midbrain_R	Thalamus_R
	Cerebellum_R	Genu of Corpus Callosum_L
	Cerebellum_L	Genu of Corpus Callosum_R
	Pons_L	Body of Corpus Callosum_L
	Pons_R	Body of Corpus Callosum_R
	AnteriorWM_L	Splenium_of Corpus Callosum_L
	AnteriorWM_R	Splenium_of Corpus Callosum_R
	PosteriorWM_L	Anterial insular cortex_L
	PosteriorWM_R	Anterial insular cortex_R
	CorpusCallosum_L	Posterial insular cortex_L
	CorpusCallosum_R	Posterial insular cortex_R
	InferiorWM_L	Lateral Ventrical_Frontal_L
	InferiorWM_R	Lateral Ventrical_body_L
	LimbicWM_L	Lateral Ventrical_atrium_L
	LimbicWM_R	Lateral Ventrical_Occipital_L
		Lateral Ventrical_Inferior_L
		Lateral Ventrical_Frontal_R
		Lateral Ventrical_body_R
		Lateral Ventrical_atrium_R
		Lateral Ventrical_Occipital_R
		Lateral Ventrical_Inferior_R
		III_and_IV_ventricle

In general, as the segmentation level goes up (fewer defined structures and more grouping voxel), the segmentation precision increases [16], because the smaller structures with volume especially less than 500 mm^3^ are more susceptible to measurement noise and errors in the segmentation pipeline. On the other hand, the location specificity decreases, as the granularity of structural definitions reduces. To evaluate the segmentation accuracy, average Dice values across the subjects based on the defined structures at each granularity level were used. One important aspect of the accuracy information that cannot be properly evaluated by these average-based reports is the occasional segmentation failures. If one out of ten cases has poor accuracy, it may not be reflected sensitively in the reports. Therefore, we also measured the accuracy by Dice ratio, which is defined as ratio of the number of cases with poor outcomes (Dice less than 0.7) and the number of all cases. The differences in Dice score due to pre-selection methods were evaluated by paired-samples T-tests. A p value < 0.01 was considered significant and p < 0.05 was considered weakly significant.

## Results

### Comparison of different pre-selection criteria as a function of the number of atlases

**Fig 4:**
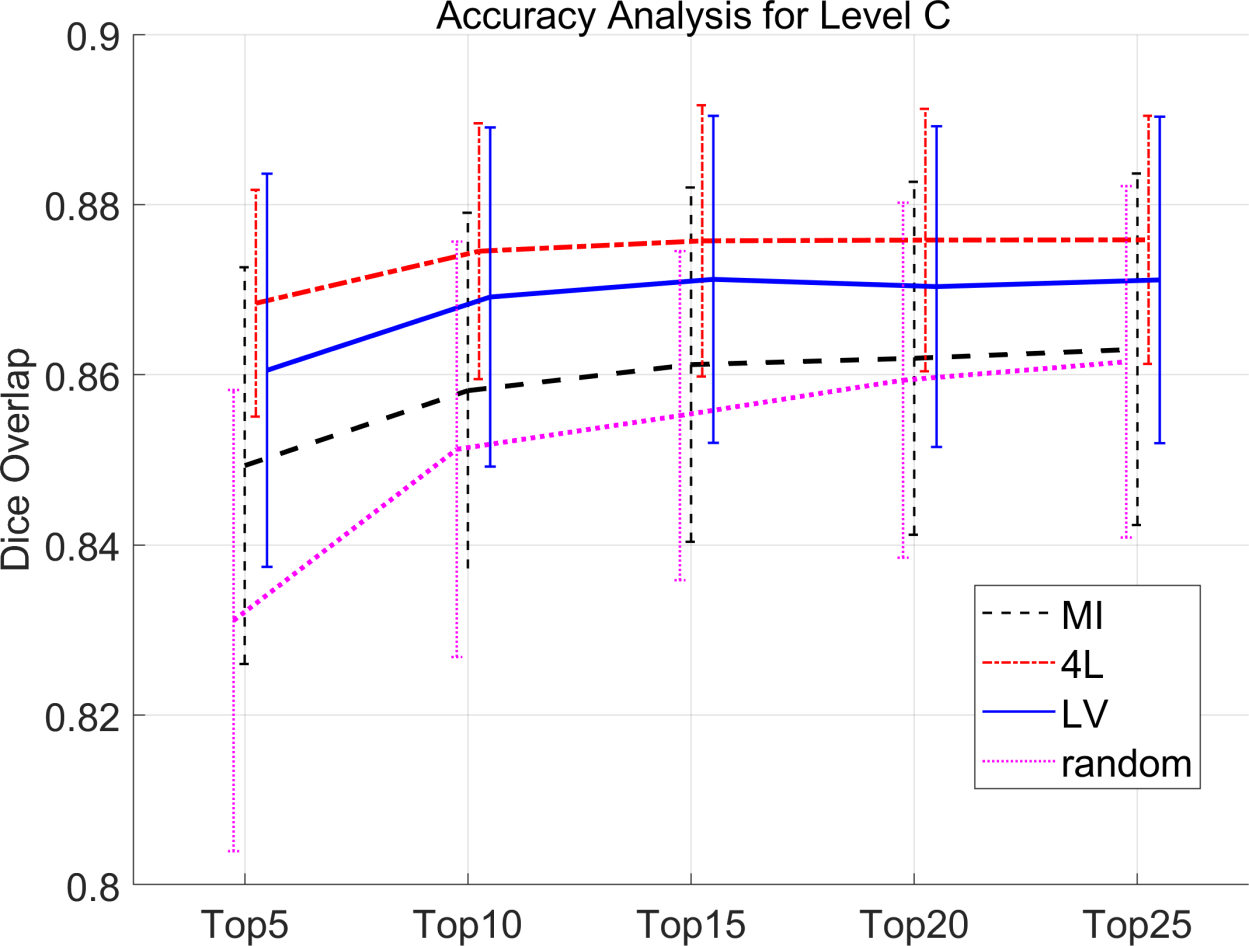
Dice overlap of Level C brain structures for all population groups with the 4L, LV, MI and random pre-selection method.

Dice values for all tests are summarized in S2 Table. In Fig 4, the results were compared for the three types of atlas selection approaches, as a function of the number of selected atlases. After the atlas ranking based on one of the three similarity measures, *k* atlases were selected for the multi-atlas segmentation. Fig 4 shows the results for the 33 structures defined at the finest granularity level (Level C). A clear trend toward accuracy improvement as a function of the number of atlases can be appreciated for all similarity criteria including the random atlas selection approach by two-way ANOVA (p<0.001). In addition, significant differences were also found among the three approaches by two-way ANOVA (p<0.001). No significant interaction was found between the atlas number and pre-selection methods.

As a function of the number of atlases, the improvement of the accuracy is steeper from 5 to 15 atlases, followed by less significant improvement, which replicated the results of previous publications [3]. The 4L and LV approaches consistently showed higher accuracy and with only 5 best atlases, accuracy comparable to 25 atlases by MI or random selections was achieved. One-way ANOVA detected differences between 4L & LV and MI & random methods (p<0.01), but there was no significant difference between the 4L and LV approaches.

### Evaluation with different age groups

**Fig 5:**
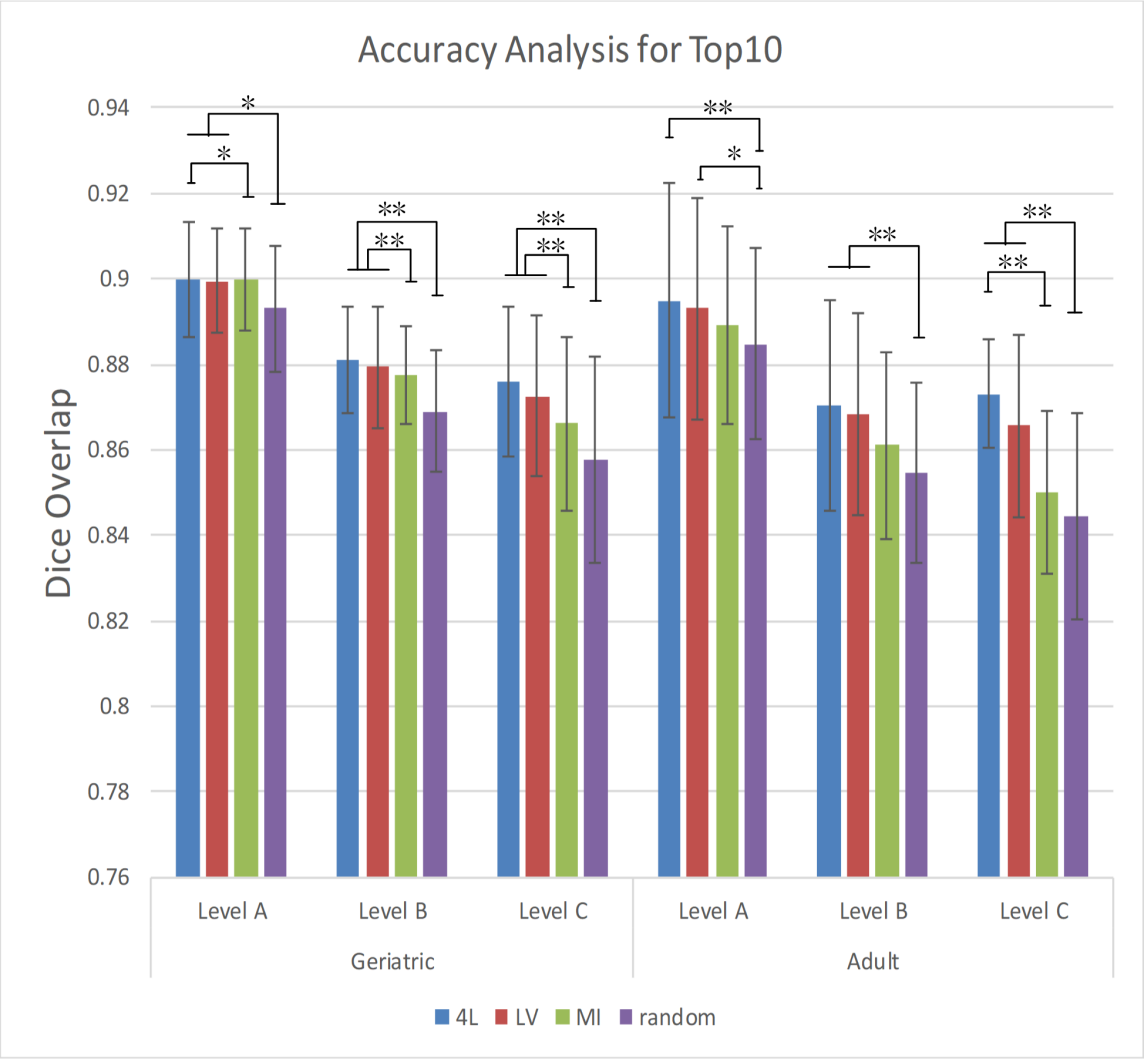
Dice overlap of Level A, Level B and Level C structures for Top 10 with the 4L, LV, MI and random pre-selection method.

Based on the top 10 atlases selected by the various approaches, the four approaches were compared using data from the geriatric and adult populations (Fig 5). S2 Table demonstrates the Dice score for all methods with different age groups and atlas numbers. For the geriatric and adult populations, all three pre-selection approaches performed equally better than the random selection while the 4L and LV-based selection consistently outperformed the MI approach. Statistical differences were found for the proposed methods compared to MI- and random pre-selection (Fig 5). The differences among the three pre-selection approaches, however, diminish for Level A and B, in which all approaches achieved remarkably high Dice levels (> 0.89).

### Analysis based on the rate of poor outcomes

**Fig 6:**
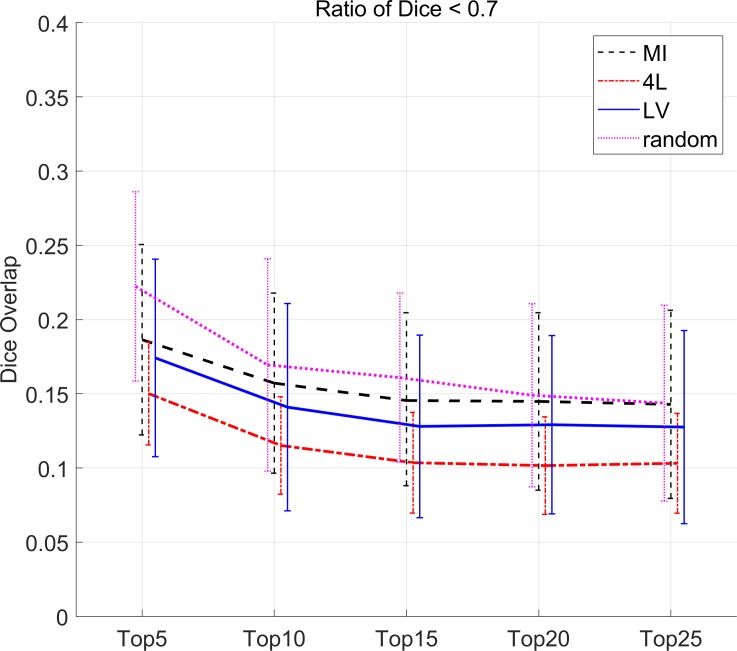
The ratio of Dice overlap < 0.7 in 286 structures for all subjects with the 4L, LV, MI and random pre-selection method.

Fig 6 shows the ratio of the segmentation results which had Dice less than 0.7. Compared to the random selections, all pre-selection approach could effectively reduce the number of poor outcomes especially when a fewer number of atlases are used. The difference became smaller when more than 20 atlases were used. Dice was shown significantly changed associated with the atlas number and pre-selection methods respectively by two-way ANOVA (p<0.001). No significant interaction was found between the atlas number and pre-selection methods. The results with 4L consistently outperformed the other approaches.

## Discussion

Recently, the technologies for the brain segmentation advanced considerably since the introduction of multiple-atlas approaches, stimulating highly active research to further enhance the accuracy and efficiency. These approaches consist of several essential components, including a multiple atlas library, accurate image registration tools, and atlas fusion algorithms. These highly promising approaches, however, have several drawbacks. First, they heavily rely on the availability of multiple atlases that define structures of interest. If a large number of structures for whole-brain segmentation are needed, atlas building becomes time-consuming and, thus, availability of the atlases could be a practical bottleneck. We recently introduced an atlas library that contains 47 atlases with 286 structures defined. The second issue is the availability of computational resources. Highly elastic image registration algorithms are computationally demanding. The multi-atlas approach requires to repeat the registration for each atlas, followed by fusion algorithms, which are also computational intensive steps. For example, the MriCloud platform employed in this study ports the data to one of the available supercomputing systems including Computational Anatomy Gateway via Extreme Science and Engineering Discovery Environment (XSEDE) (http://info.teragrid.org/web-apps/html/views/tggateways) and, yet, the calculation with 20 atlases takes approximately 30 mins. Usage of more atlases would further increase the burden to computational resources.

For atlas pre-selection, image feature matching had been tested in the past including the mutual information-based approach tested in this paper. One potential limitation of the image-intensity based approach is lack of location information, which only considers image-intensity features of the entire tissue included in the image. The accuracy of the feature matching could be improved by defining the brain tissue and including only the voxels inside the brain. By further introducing location information through image segmentation, more detailed anatomical features can be extracted. This led to the idea of performing quick and macroscopic segmentation of the brain into the several basic anatomical components (gray matter, white matter, ventricles, and CSF space) and using them for the location-based feature matching.

In this study, the initial registration was performed by linear transformation followed by the likelihood fusion, with which initial segmentation with 46 atlases completed in approximately 5 min using the XSEDE computation resources. Once appropriate atlases were selected, the main segmentation algorithm with highly elastic image transformation and full 286 label calculation took approximately 20 min using 10 selected atlases. Compared to using all 46 atlases for the full brain segmentation, the calculation time was shortened by more than 1 hour. This difference increases as the larger atlas inventories are searched for pre-selection. The calculation for the 4L- and LV-based image similarity measure can be fulfilled very fast on XSEDE server. However, the computation for MI of a paired brain T1 image can be 20 times that of the proposed pre-selection measures. Given a database with 10 atlases, the 4L- and LV-based approaches would take less than 1 min, in comparison with 19.2 min when using MI-based pre-selection. Therefore, our proposed atlas pre-selection strategies have significant advantage on the computation efficiency over the traditional one.

**Fig 7:**
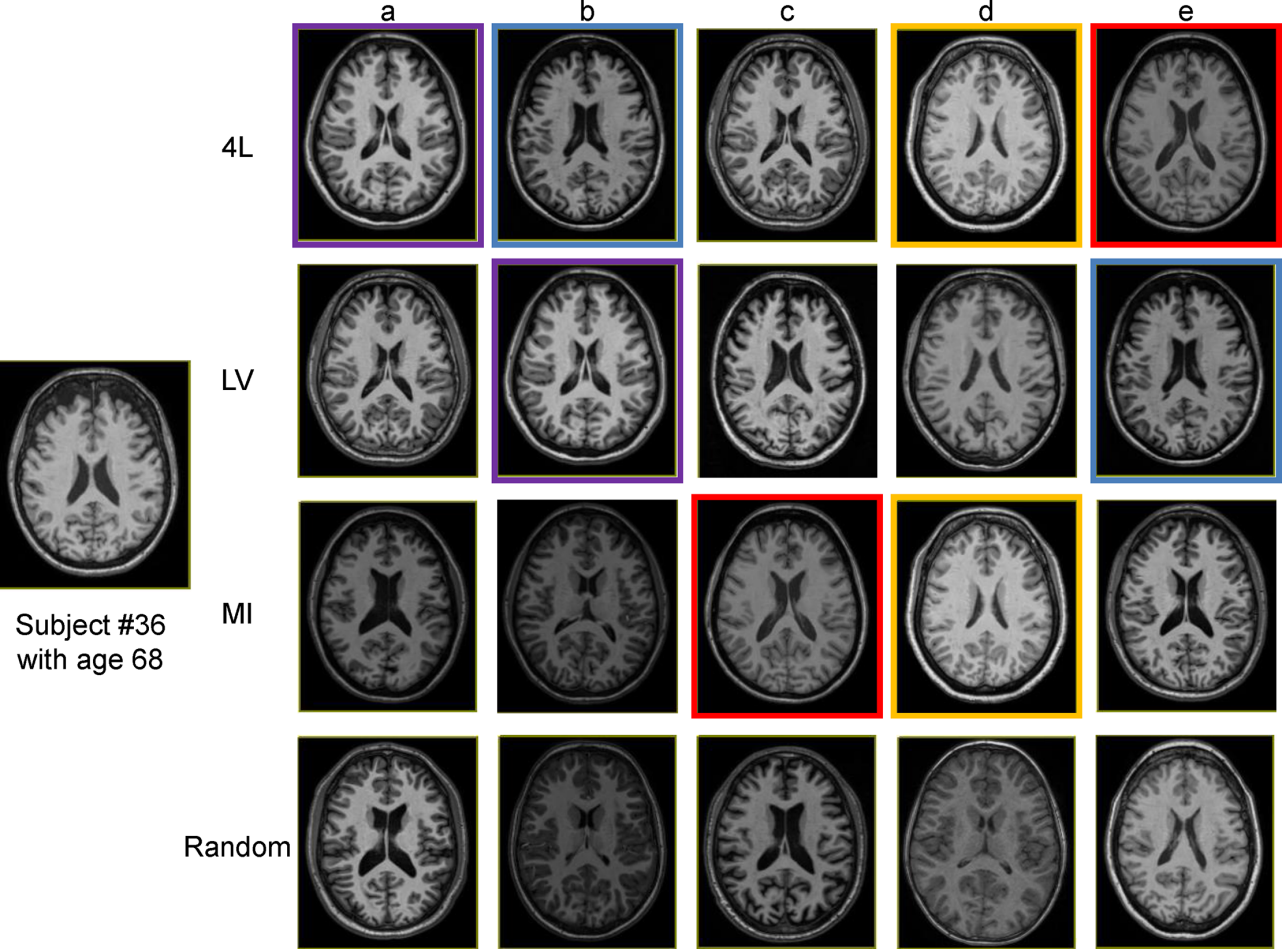
Examples of 5 selected atlases for a geriatric subject with different atlas pre-selection approaches. The same atlases shared across different pre-selection approaches are indicated with the same border color.

**Fig 8:**
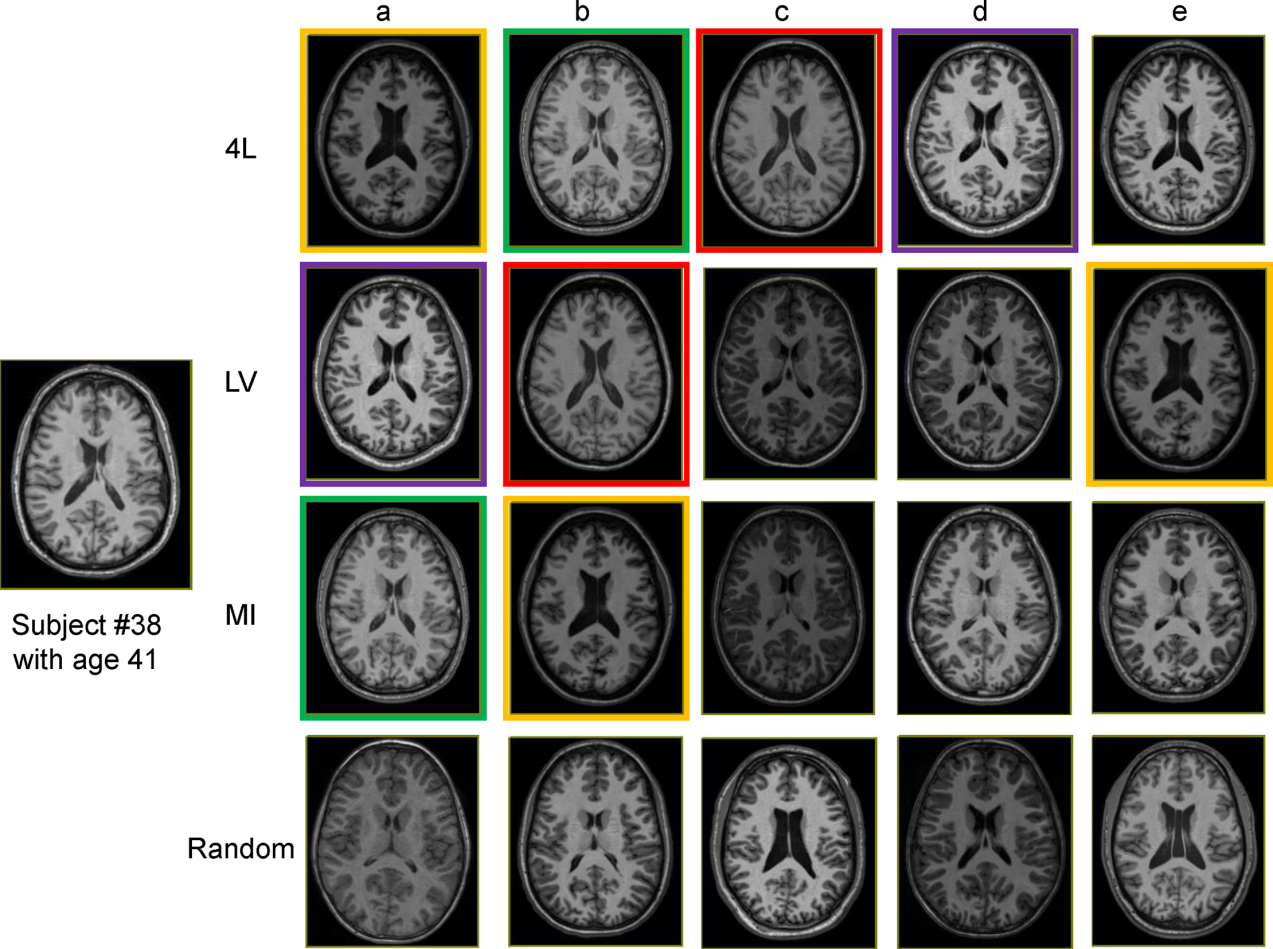
Examples of 5 selected atlases for an adult subject with different atlas pre-selection approaches. The same atlases shared across different pre-selection approaches are indicated with the same border color.

The quantitative analyses based on Dice overlap showed the improved accuracy by all the three methods, while the 4L approach consistently led to the highest level of accuracy at a given number of employed atlases. Alternatively, a given accuracy level could be achieved with a smaller number of atlases by employing the atlas pre-selection (Fig 4). Analyses with different age groups showed the improvement was found in both geriatric and young adult populations (Fig 5). Out of the four defined levels, the ventricle label seemed to have the highest impact on the improvement of the accuracy level, which is in good agreement with previous publications [3, 13] This is understandable because the ventricle, one of the most difficult objects to register across subjects, has a large amount of variability in their shapes among populations. Choosing atlases with similar ventricle shapes would lead to more accurate structure-to-structure registrations. Figs 7 and 8 show actual images selected for subjects at different ages based on four different criteria. The variation of volume and morphology of the five selected atlases appears greater with 4L and LV method than MI and random method, especially for lateral ventricle. Interestingly, the selected five atlases with 4L approach share some cases that can be found in the selected atlases with either LV or MI approach for both the geriatric subject (Fig 7A for 4L = Fig 7B for LV, Fig 7B for 4L = Fig 7E for LV, Fig 7D for 4L = Fig 7D for MI, Fig 7E for 4L = Fig 7C for MI) and the adult subject (Fig 8A for 4L = Fig 8E for LV = Fig 8B for MI, Fig 8B for 4L = Fig 8A for MI, Fig 8C for 4L = Fig 8B for LV, Fig 8D for 4L = Fig 8A for LV). Thus it can be appreciated that the 4L approach successfully identified subjects with similar anatomy qualitatively.

The rate of the poor outcomes (Fig 6) also indicated fewer rates by the 4L approach. Please note that even with the 4L approach and more than 20 atlases, there are still more than 10% of the results with poor (Dice < 0.7) outcomes. This is likely due to the fact that there were not enough atlases with similar anatomy within the employed atlas library. Ten percentile outliers have only 2 atlases in average that would have similar anatomy with 20 atlases. This could be improved by a larger number of atlases or better registration algorithms, but further studies are needed to test this hypothesis.

In conclusion, we have proposed a new atlas pre-selection strategy for multi-atlas brain segmentation based on a structure-to-structure similarity measure. Such method would provide better segmentation performance in terms of both accuracy and efficiency. The proposed atlas pre-selection will be further implemented into our online automatic brain image segmentation system (www.mricloud.org).

## Supporting information

S1 Table The hierarchical relationships of different granularity levels (Level A, Level B and Level C). (DOCX)

S2 Table Dice overlap of selected regions for three age groups by different atlas pre-selection methods. (DOCX)
