# Supplementary material for: Atlas pre-selection strategies to enhance the efficiency and accuracy of multi-atlas brain segmentation tools
Source: PLoS One. 2018 Jul 27;13(7):e0200294. doi: 10.1371/journal.pone.0200294 (PMC6063392; doi:10.1371/journal.pone.0200294)
Supplement: S1 Table — (DOCX) [file pone.0200294.s001.docx]

**S1 Table:** **The hierarchical relationships of different granularity levels (Level A, Level B and Level C).**

| **Level C** | **Level B** | **Level A** |
| --- | --- | --- |
| Superior Frontal Gyrus_L | Frontal_L | CerebralCortex_L |
| Superior Frontal Gyrus_R | Frontal_R | CerebralCortex_R |
| Middle Frontal Gyrus_L | Frontal_L | CerebralCortex_L |
| Middle Frontal Gyrus_R | Frontal_R | CerebralCortex_R |
| Inferior Frontal Gyrus_L | Frontal_L | CerebralCortex_L |
| Inferior Frontal Gyrus_R | Frontal_R | CerebralCortex_R |
| Fronto-orbital Gyrus_L | Frontal_L | CerebralCortex_L |
| Fronto-orbital Gyrus_R | Frontal_R | CerebralCortex_R |
| Rectus Gyrus_L | Frontal_L | CerebralCortex_L |
| Rectus Gyrus_R | Frontal_R | CerebralCortex_R |
| Postcentral Gyrus_L | Parietal_L | CerebralCortex_L |
| Postcentral Gyrus_R | Parietal_R | CerebralCortex_R |
| Precentral Gyrus_L | Frontal_L | CerebralCortex_L |
| Precentral Gyrus_R | Frontal_R | CerebralCortex_R |
| Superior Parietal Gyrus_L | Parietal_L | CerebralCortex_L |
| Superior Parietal Gyrus_R | Parietal_R | CerebralCortex_R |
| Supramarginal Gyrus_L | Parietal_L | CerebralCortex_L |
| Supramarginal Gyrus_R | Parietal_R | CerebralCortex_R |
| Angular Gyrus_L | Parietal_L | CerebralCortex_L |
| Angular Gyrus_R | Parietal_R | CerebralCortex_R |
| Precuneus_L | Parietal_L | CerebralCortex_L |
| Precuneus_R | Parietal_R | CerebralCortex_R |
| Superior Temporal Gyrus_L | Temporal_L | CerebralCortex_L |
| Superior Temporal Gyrus_R | Temporal_R | CerebralCortex_R |
| Middle Temporal Gyrus_L | Temporal_L | CerebralCortex_L |
| Middle Temporal Gyrus_R | Temporal_R | CerebralCortex_R |
| Inferior Temporal Gyrus_L | Temporal_L | CerebralCortex_L |
| Inferior Temporal Gyrus_R | Temporal_R | CerebralCortex_R |
| Limbic_L | Limbic_L | CerebralCortex_L |
| Limbic_R | Limbic_R | CerebralCortex_R |
| Fusiform Gyrus_L | Temporal_L | CerebralCortex_L |
| Fusiform Gyrus_R | Temporal_R | CerebralCortex_R |
| Superior Occipital Gyrus_L | Occipital_L | CerebralCortex_L |
| Superior Occipital Gyrus_R | Occipital_R | CerebralCortex_R |
| Middle Occipital Gyrus_L | Occipital_L | CerebralCortex_L |
| Middle Occipital Gyrus_R | Occipital_R | CerebralCortex_R |
| Infeiror Occipital Gyrus_L | Occipital_L | CerebralCortex_L |
| Infeiror Occipital Gyrus_R | Occipital_R | CerebralCortex_R |
| Cuneus_L | Occipital_L | CerebralCortex_L |
| Cuneus_R | Occipital_R | CerebralCortex_R |
| Lingual Gyrus_L | Occipital_L | CerebralCortex_L |
| Lingual Gyrus_R | Occipital_R | CerebralCortex_R |
| Cingulate_L | Limbic_L | CerebralCortex_L |
| Cingulate_R | Limbic_R | CerebralCortex_R |
| Anterial Insular Cortex_L | Insula_L | CerebralCortex_L |
| Anterial Insular Cortex_R | Insula_R | CerebralCortex_R |
| Posterial Insular Cortex_L | Insula_L | CerebralCortex_L |
| Posterial Insular Cortex_R | Insula_R | CerebralCortex_R |
| Amygdala_L | Limbic_L | CerebralNucli_L |
| Amygdala_R | Limbic_R | CerebralNucli_R |
| Hippocampus_L | Limbic_L | CerebralCortex_L |
| Hippocampus_R | Limbic_R | CerebralCortex_R |
| Caudate_L | BasalGang_L | CerebralNucli_L |
| Caudate_R | BasalGang_R | CerebralNucli_R |
| Putamen_L | BasalGang_L | CerebralNucli_L |
| Putamen_R | BasalGang_R | CerebralNucli_R |
| Globus pallidus_L | BasalGang_L | CerebralNucli_L |
| Globus pallidus_R | BasalGang_R | CerebralNucli_R |
| Thalamus_L | Thalamus_L | Thalamus_L |
| Thalamus_R | Thalamus_R | Thalamus_R |
| midbrain_L | midbrain_L | Mesencephalon_L |
| midbrain_R | midbrain_R | Mesencephalon_R |
| Cerebellum_R | Cerebellum_R | Metencephalon_R |
| Cerebellum_L | Cerebellum_L | Metencephalon_L |
| Pons_L | Pons_L | Metencephalon_L |
| Pons_R | Pons_R | Metencephalon_R |
| Genu of Corpus Callosum_L | CorpusCallosum_L | WhiteMatter_L |
| Genu of Corpus Callosum_R | CorpusCallosum_R | WhiteMatter_R |
| Body of Corpus Callosum_L | CorpusCallosum_L | WhiteMatter_L |
| Body of Corpus Callosum_R | CorpusCallosum_R | WhiteMatter_R |
| Splenium_of Corpus Callosum_L | CorpusCallosum_L | WhiteMatter_L |
| Splenium_of Corpus Callosum_R | CorpusCallosum_R | WhiteMatter_R |
| PosteriorWM_L | PosteriorWM_L | WhiteMatter_L |
| PosteriorWM_R | PosteriorWM_R | WhiteMatter_R |
| InferiorWM_L | InferiorWM_L | WhiteMatter_L |
| InferiorWM_R | InferiorWM_R | WhiteMatter_R |
| LimbicWM_L | LimbicWM_L | WhiteMatter_L |
| LimbicWM_R | LimbicWM_R | WhiteMatter_R |
| AnteriorWM_L | AnteriorWM_L | WhiteMatter_L |
| AnteriorWM_R | AnteriorWM_R | WhiteMatter_R |
| CerebellumWM_R | Cerebellum_R | Metencephalon_R |
| CerebellumWM_L | Cerebellum_L | Metencephalon_L |
| Lateral Ventrical_Frontal_L | LateralVentricle_L | Ventricle |
| Lateral Ventrical_body_L | LateralVentricle_L | Ventricle |
| Lateral Ventrical_atrium_L | LateralVentricle_L | Ventricle |
| Lateral Ventrical_Occipital_L | LateralVentricle_R | Ventricle |
| Lateral Ventrical_Inferior_L | LateralVentricle_R | Ventricle |
| Lateral Ventrical_Frontal_R | LateralVentricle_R | Ventricle |
| Lateral Ventrical_body_R | LateralVentricle_R | Ventricle |
| Lateral Ventrical_atrium_R | LateralVentricle_R | Ventricle |
| Lateral Ventrical_Occipital_R | LateralVentricle_R | Ventricle |
| Lateral Ventrical_Inferior_R | LateralVentricle_R | Ventricle |
| III_ventricle | III_ventricle | Ventricle |
| IV_ventricle | IV_ventricle | Ventricle |
