# Supplementary material for: Atlas pre-selection strategies to enhance the efficiency and accuracy of multi-atlas brain segmentation tools
Source: PLoS One. 2018 Jul 27;13(7):e0200294. doi: 10.1371/journal.pone.0200294 (PMC6063392; doi:10.1371/journal.pone.0200294)
Supplement: S2 Table — (DOCX) [file pone.0200294.s002.docx]

**S2 Table: Dice overlap of selected regions for three age groups by different atlas pre-selection methods.**

|  |  |  | 4L | LV | MI | Random |
| --- | --- | --- | --- | --- | --- | --- |
| Geriatric | Top 5 | Level A | 0.885±0.026 | 0.883±0.025 | 0.879±0.023 | 0.870±0.022 |
|  |  | Level B | 0.861±0.025 | 0.859±0.024 | 0.852±0.022 | 0.839±0.023 |
|  |  | Level C | 0.868±0.011 | 0.859±0.195 | 0.843±0.022 | 0.828±0.178 |
|  | Top 10 | Level A | 0.895±0.274 | 0.893±0.026 | 0.889±0.023 | 0.885±0.022 |
|  |  | Level B | 0.870±0.024 | 0.869±0.024 | 0.861±0.022 | 0.855±0.021 |
|  |  | Level C | 0.873±0.013 | 0.866±0.021 | 0.850±0.019 | 0.845±0.024 |
|  | Top 15 | Level A | 0.897±0.027 | 0.896±0.027 | 0.892±0.024 | 0.887±0.022 |
|  |  | Level B | 0.872±0.025 | 0.871±0.024 | 0.863±0.022 | 0.858±0.020 |
|  |  | Level C | 0.871±0.013 | 0.866±0.019 | 0.852±0.020 | 0.850±0.018 |
|  | Top 20 | Level A | 0.898±0.027 | 0.897±0.026 | 0.892±0.024 | 0.892±0.024 |
|  |  | Level B | 0.873±0.025 | 0.873±0.025 | 0.864±0.022 | 0.863±0.022 |
|  |  | Level C | 0.872±0.013 | 0.865±0.021 | 0.851±0.019 | 0.849±0.020 |
|  | Top 25 | Level A | 0.899±0.026 | 0.898±0.026 | 0.894±0.024 | 0.894±0.025 |
|  |  | Level B | 0.874±0.024 | 0.873±0.024 | 0.865±0.022 | 0.866±0.023 |
|  |  | Level C | 0.872±0.011 | 0.865±0.020 | 0.852±0.017 | 0.853±0.018 |
| Adult | Top 5 | Level A | 0.892±0.012 | 0.890±0.011 | 0.890±0.012 | 0.875±0.020 |
|  |  | Level B | 0.873±0.011 | 0.868±0.014 | 0.868±0.013 | 0.849±0.023 |
|  |  | Level C | 0.869±0.016 | 0.862±0.027 | 0.855±0.024 | 0.834±0.035 |
|  | Top 10 | Level A | 0.900±0.014 | 0.899±0.012 | 0.900±0.012 | 0.893±0.015 |
|  |  | Level B | 0.881±0.012 | 0.879±0.014 | 0.877±0.011 | 0.869±0.014 |
|  |  | Level C | 0.876±0.018 | 0.873±0.019 | 0.866±0.204 | 0.859±0.024 |
|  | Top 15 | Level A | 0.903±0.013 | 0.902±0.011 | 0.903±0.012 | 0.895±0.013 |
|  |  | Level B | 0.884±0.013 | 0.882±0.014 | 0.881±0.012 | 0.871±0.013 |
|  |  | Level C | 0.880±0.018 | 0.877±0.018 | 0.870±0.019 | 0.860±0.020 |
|  | Top 20 | Level A | 0.905±0.013 | 0.902±0.010 | 0.904±0.013 | 0.900±0.011 |
|  |  | Level B | 0.886±0.013 | 0.881±0.011 | 0.882±0.013 | 0.877±0.010 |
|  |  | Level C | 0.880±0.018 | 0.876±0.016 | 0.872±0.018 | 0.870±0.017 |
|  | Top 25 | Level A | 0.905±0.013 | 0.903±0.010 | 0.905±0.012 | 0.899±0.012 |
|  |  | Level B | 0.886±0.013 | 0.882±0.012 | 0.883±0.013 | 0.878±0.012 |
|  |  | Level C | 0.880±0.017 | 0.877±0.017 | 0.874±0.018 | 0.870±0.020 |
|  |  | Level C | 0.858±0.031 | 0.861±0.028 | 0.859±0.030 | 0.855±0.028 |
